# Building brain-inspired computing

**DOI:** 10.1038/s41467-019-12521-x

**Published:** 2019-10-18

## Abstract

Dmitri Strukov (an electrical engineer, University of California at Santa Barbara), Giacomo Indiveri (an electrical engineer, University of Zurich), Julie Grollier (a material physicist, Unite Mixte de Physique CNRS) and Stefano Fusi (a neuroscientist, Columbia University) talked to *Nature Communications* about the opportunities and challenges in developing brain-inspired computing technologies, namely neuromorphic computing, and advocated effective collaborations crossing multidisciplinary research areas to support this emerging community.

**Figure:**
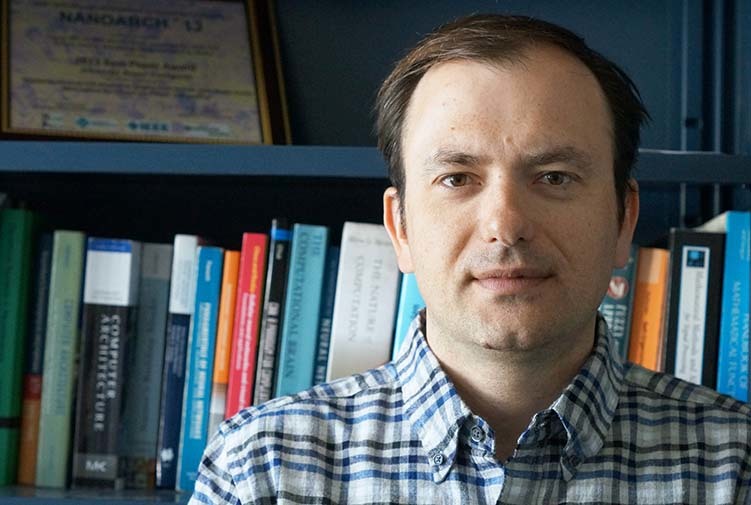
Dmitri Strukov

1. Please tell us about your research background and how it brought you to work on neuromorphic computing?

**DS**: I was trained as an electrical engineer and got interested in developing circuits and architectures using emerging electron devices in my graduate school at Stony Brook University. Afterwards, I moved to Hewlett Packard Laboratories as a postdoctoral researcher and switched my attention to device physics. I spent most of my time developing models for mixed electronic-ionic conductors that could be used to implement resistive switching devices (known as memristors nowadays). This experience naturally led me to choose neuromorphic computing—one of the most promising applications of memristors—as my research area after I joined University of California at Santa Barbara. My major focus now is on the development of practical mixed-signal circuits for artificial neural networks. This is a challenging topic because it spans across a broad range of disciplines, from electron devices to algorithms. In the long term, I hope that our research will lead to practically useful neuromorphic systems that will be used in everyday life.

**Figure:**
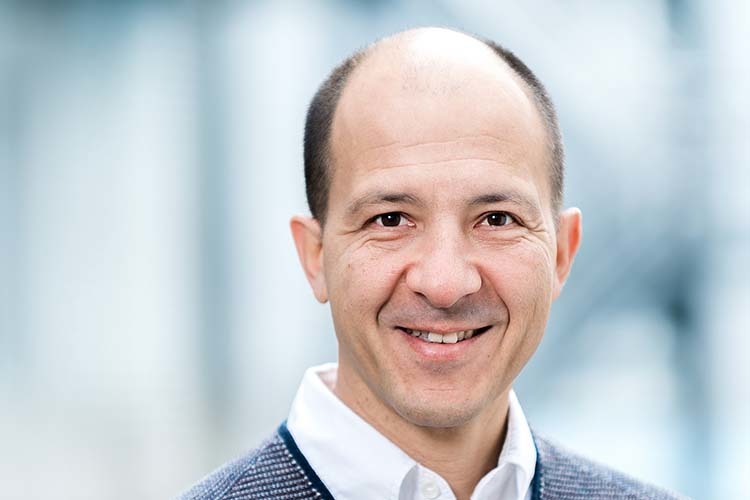
Giacomo Indiveri

**GI**: I am an electrical engineer by training, but started to get interested in bio-physics and neural computation since my graduate school. In my Master’s thesis, I developed a model of early vision processing using recurrent networks of simple and complex visual cells. By attending courses of John Hopfield and Federico Faggin within a national program on bio-technologies in Italy, I learned about neural networks and their microelectronic implementation. Fascinated by this research area, I joined the lab of Christof Koch (the current Chief Scientific Officer at the Allen Institute) at Caltech in 1994. It was there that I learned much more about the biophysics of neural computation and about neuromorphic electronic circuits—also through interactions with the members of Carver Mead’s lab and via his lectures. Since then, I’ve been hooked on research that involves the design of elegant analog subthreshold electronic circuits that emulate synaptic and neural dynamics. The aim is to understand the computational principles used by real neurons on cortical circuits.

**Figure:**
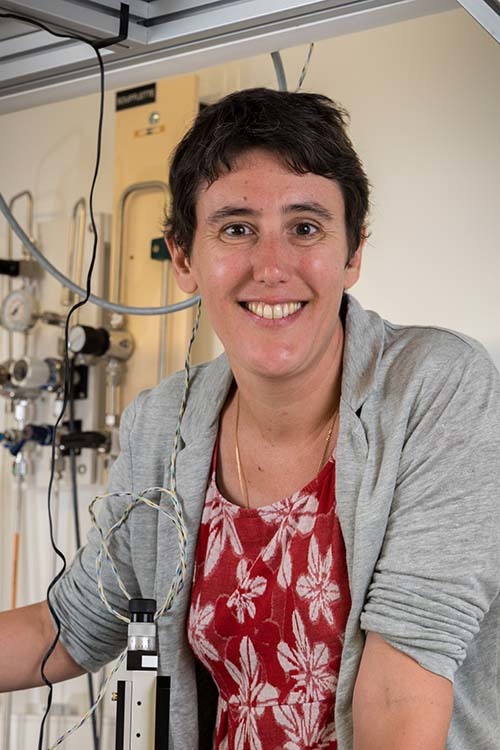
Julie Grollier

**JG**: I’m a condensed matter physicist. Since the beginning of my career, I’ve been working in the field of spintronics. One day in 2009, while leafing through a science magazine, I stumbled on an article about memristors. The paper was explaining that these nanodevices could emulate the synapses of the brain. That was so fascinating, I was enthralled. It made me wonder if spintronics could be useful for brain-inspired computing as well. And actually I think it is! Recently, we have shown that magnetic tunnel junctions can show synapse- and neuron-like functionalities, and even perform pattern recognition. Now we’re trying to figure out how to connect many single devices together to build integrated circuits and make them compute in an efficient way.

**Figure:**
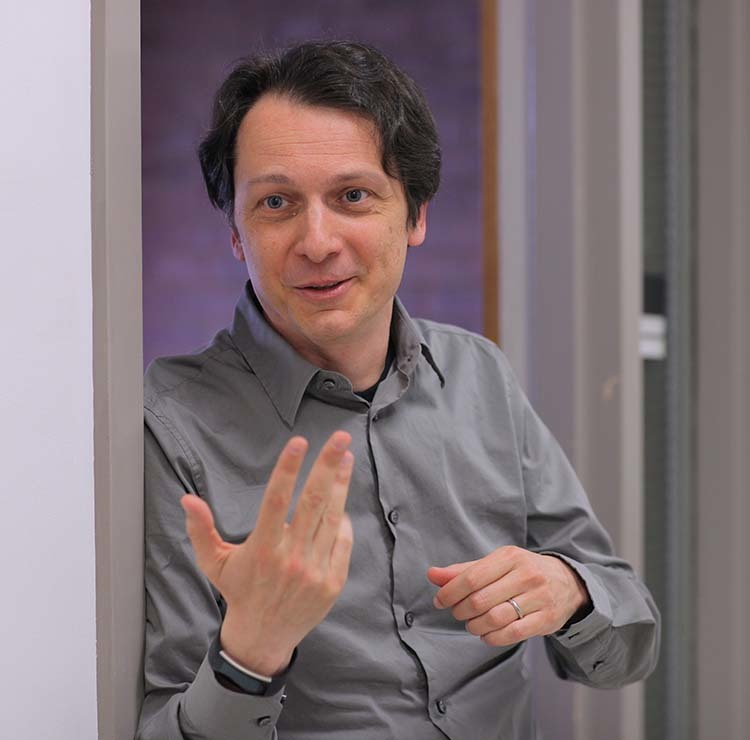
Stefano Fusi

**SF**: My background is theoretical physics but very early in my scientific career I got interested in neuromorphic computing because of a project of the National Institute of Nuclear Physics in which my advisor Daniel J. Amit and I were involved. The project was aimed at classifying particle traces in real time using neural networks. I realized immediately that unlike particle physics, the neural network theory was still in the early days and most of the important computational principles were poorly understood or had to be discovered. That is why I switched to neuroscience, and in particular I got interested in learning about synaptic plasticity. At that time, demonstrating online learning in a neuromorphic device was still a challenge. I was hoping I could take inspiration from the biological brain to understand how to design a new generation of energy-efficient learning devices.

2. Why do we need neuromorphic computing?

**DS**: The answer is quite obvious if one interprets neuromorphic computing as a biologically inspired computing technology facilitated by powerful deep learning algorithms that have already showed profound impact on science, technology, and our society. However, when considering the very original definition of neuromorphic computing coined by Carver Mead at Caltech, which can be loosely put as “analog computing hardware organized similarly to the brains”, the answer becomes less clear to me. This is in part because such definition still leaves some ambiguity in how closely neuromorphic computing hardware should emulate the brains and what functionalities are expected from such systems. One could call neuromorphic computing a hardware that is merely borrowing a few tricks from biology, such as perceptron-like distributed parallel information processing, to perform simple machine learning tasks. Conversely, should it also integrate more advanced functions (e.g. spike-time encoding, various types of plasticity, homeostasis, etc.) and be capable of realizing cognitive functions at higher levels? Nevertheless, the primary motivation is arguably to achieve the extreme energy efficiency of the brains using neuromorphic computing. In fact, this will be the main advantage of analog and mixed-signal implementations of simple perceptron networks as well as of advanced spiking neural networks. Some existing results, albeit they perform simple tasks like image classification, have shown many orders of magnitudes improvement in energy and speed compared to purely digital computing, and some of them can even surpass the performance of the human brain.

**GI**: The basic research activities in neuromorphic computing that we do at the Institute of Neuroinformatics are aimed at ‘understanding by building’. The inspiration for this research comes from Caltech and specifically from the quote that was found on Richard Feynman’s blackboard “What I Cannot Create, I Do Not Understand”. By operating transistor channels and transistors in a region that follows the same physics of protein channels across neuron membranes, we can make amazing analogies between neural networks in brains and complex circuits that use such transistors to emulate neuron and synapse dynamics. Ideally, by building artificial neural processing systems that are governed by the same physics and are constrained by the same factors, such as limited resolution, low signal-to-noise ratios, and variable or inhomogeneous processing elements, we can better understand why certain cortical circuits in the brain are configured and operate in certain ways. Another benefit lies in the extremely low power consumption at sub-milli-watt level for the operation of these neuromorphic systems, making them a very attractive approach for building ultra-low power neural processing systems that can complement and aid more conventional computing technologies.

**JG**: Artificial intelligence (AI) needs new hardware, not just new algorithms. We’re at a turning point, where Moore’s law is reaching its end leading to a stagnation of the performance of our computers. Nowadays, we are generating more and more data that needs to be stored and classified. The recent progresses in AI allow automating this process, with data centers multiplying at a cost of consuming an exponentially increasing amount of electricity a potential problem for our environment. This energy consumption mainly comes from data traffic between memory and processing units that are separated in computers. It wastes electrical energy and it considerably slows down computational speed. Recent developments in nanotechnology offer the possibility to bring huge amounts of memory close to processing, or even better, to integrate this memory directly in the processing unit. The idea of neuromorphic computing is to take inspiration of the brain for designing computer chips that merge memory and processing. In the brain, synapses provide a direct memory access to the neurons that process information. That’s how the brain achieves impressive computational power and speed with very little power consumption. By imitating this architecture, neuromorphic computing provides a path to building smart chips that consume very little energy and, meanwhile, compute fast.

3. What can we learn from our brain for information processing? How to emulate human brain using electronic devices and where are we now?

**DS**: There is a general consensus on the usefulness of some tricks that are employed by the brains, such as analog and in-memory computing, massively parallel processing, spike coding, task-specific connectivity in neural networks. Many of these ideas have already been implemented in state-of-the-art neuromorphic systems. I do believe, however, that we should not blindly try to mimic all features of the brains—at least not doing so without having a good engineering reason first—and we should consider simpler approaches based on more conventional technologies to achieve the same goal. On the other hand, we should also keep in mind that over millions of years the evolution of biological brains has been constrained to biomaterials optimized for specific tasks, while we have a much wider range of material choices now in the context of neuromorphic engineering. Therefore, there could exist profound differences in designing rules. For example, the brains have to rely on poor conductors offered by biomaterials, which have presumably affected the principles of brain structure and operation in some ways that are not necessarily to be applicable to neuromorphic computing based on high conducting materials.

**GI**: Neuroscience has made tremendous progress in uncovering information processing principles used by the brains. The machine learning and artificial neural network community has already applied the principles of visual and auditory processing to image, video, sound and speech recognition. Now, the community is expanding the principles of unsupervised and reinforcement learning to solve a wider range of practical problems (see the successes of DeepMind as an example). Up to now, most of these successes have been at the theoretical and algorithmic level using a computing substrate that is completely detached from the algorithmic one (i.e., standard computers based on the von Neumann architecture and on the Turing Machine framework). There is a great effort now in trying to understand how to implement new types of algorithms using novel computing architectures that are inspired by the way real nervous systems organize and carry out computation. To this end a radical departure from the existing computing technologies and innovation at all levels are required, ranging from single memory devices to full-scale in-memory computing architectures that combine analog with digital processing elements for both computation and signal transmission. Fortunately, we have many different types of brains as a great source of inspiration for these innovations. We can start from small and simple insect brains to understand how to build efficient (i.e., low power and low volume) neural computing hardware architectures that go beyond von Neumann computing technologies.

**JG**: We don’t completely understand how the brain is working, but we know that it is made very differently from today’s electronic chips. The brain is constituted of synapses and neurons instead of memory blocks and transistors. It stores information in an analog way and not in bits. Its components are noisy and not of high precision. It displays multiple dynamics instead of having a single clock. It is plastic instead of having limited reconfigurability. The current computer processors rely on semiconductor physics and compute through the laws of electricity. All these fundamental differences between brains and chips could indicate that other physical principles might turn out to be more useful to emulate the brain behavior. That is what people are investigating today.

**SF**: Our brain consumes as little as 20 Watts. If we understand the architectural and computational principles of biological brains, we might be able to significantly reduce the energy consumption of our electronic devices. There are several prominent features of the biological brains that we still do not understand, and hence are usually not implemented in artificial neural networks used in real-world applications. One is the laminar structure, which is observed in the cortex of many species. Although there are some ideas about the computational role of the different layers, state-of-the-art deep neural networks seem to work perfectly well without a laminar architecture. Laminarity can be easily implemented, but does not seem to improve much the performance. A second feature is the huge diversity observed in biological brains. For example, there are several different types of easily distinguishable neuronal cells, whereas in most deep neural networks all the neurons are identical. Any attempt to introduce biological diversity can either disrupt the performance or lead to a very modest improvement. A third feature is the high degree of organization of biological brains. It is possible to identify seemingly specialized ‘areas’ that, for example, tend to encode information about faces. This organization is preserved across individuals but it is rarely considered in deep networks. Finally, and most importantly, the synapses in biological systems are rather complicated networks of biochemical processes, whereas in artificial neural networks are typically encoded by a single variable that represent the synaptic weight (see also question 5). All these differences might be due to the fact that the neural networks implemented by our brain and those implementable in electronic devices use very different ‘hardware’. However, it is also possible that understanding the computational role of all these biological features will tell us how to design more efficient artificial neural networks.

4. How to best utilize the existing science and technology we learn from the conventional electronics to develop neuromorphic computing?

**GI**: It is commonly agreed that if the nervous system is required to achieve a desired goal, it uses multiple mechanisms in parallel to take advantage of all the resources it has at hand. I believe the same principle should be applied to building neuromorphic systems. Of course there will be different constraints that will restrict some of the potential approaches that can be followed (e.g. time-to-market constraints might favor the use of standard synchronous design-flows compared to asynchronous or mixed signal analog-digital ones). But as a guiding principle, a good neuromorphic engineer should try to use the best of all the existing or/and emerging technologies and tools that exist today to optimize the design of the targeted neuromorphic computing system.

**JG**: Propagating information in densely connected and parallel systems such as neural networks requires signal gain. I believe that transistors will remain the best way to provide signal gain for many years because this is something they do very well (industry optimized them for this purpose since the last century). So, it is very likely that future neuromorphic chips will use transistors to route electricity towards novel nanodevices connected in a network. The trick will be to design these systems in the most efficient way and to reduce as much as possible the circuit area and energy consumption of transistor-based interconnects.

5. What are the major hurdles to date towards realizing neuromorphic computing from your perspective?

**DS**: In my opinion, there are tough challenges at several levels. From a technology perspective, the foremost challenge is various device non-idealities, such as the notorious device-to-device variations in their current-voltage characteristics and poor yields of memory devices—one of the key components of neuromorphic circuits (I will elaborate more on these issues in the answer to question 6). In addition to these technological hurdles, I reckon that there might be other substantial economical and confidence barriers to achieve such highly innovative, yet high-risk technology. Ultimately, to be successful, neuromorphic computing hardware would have to win competition over conventional digital circuits that are supported by presently available infrastructures and enormous investments over years. Fortunately, this barrier does not appear to be as bad as, say, 20 years ago, because of slowing down innovations (mainly about feature size scaling) in conventional CMOS technology, very high development and production cost of sub-10-nm CMOS circuits, and general trend towards more specialized computing hardware. Apart from hardware issues, the progress on the algorithmic front is clearly not sufficient to cope with the explosive increase in the need from neuromorphic computing either, especially for higher cognition tasks. The lack of suitable algorithms, in return, has imposed large uncertainty in designing neuromorphic hardware.

**GI**: The construction of an optimal neuromorphic computing system requires knowledge of fundamental neuroscience, computer science, materials science, robotics, microelectronics as well as creativity and insights. The major hurdle is in acquiring all these interdisciplinary notions, or (from a university professor’s point of view) in training the next generation of neuromorphic engineers that have this broad set of competences and skills.

**JG**: The devil is in the numbers. Neural networks need huge amounts of synapses and neurons to be computationally powerful. There are a hundred billion neurons and ten thousand times more synapses in our brains. If we want to build small chips with such huge number of elements, we are going to need nanoneurons and nanosynapses. So, the first challenge is to imitate important functions of synapses and neurons, such as long-term memory, nonlinearity or spiking behaviour, using devices at nanoscales. We need new physics and new materials to achieve this goal. The second challenge is wiring. The brain is like a crazy three-dimensional wool ball with interconnected organic wires. Each neuron is connected in average of ten thousand synapses. How can we artificially achieve such degrees of interconnection? This is going to be further complicated by our currently available electronics because at the moment it is largely two dimensional and made of regular grids of wires.

**SF**: The human brain contains 10^14^−10^15^ synapses (most of them are probably plastic), which means that they are continuously modified to acquire new information, store memories or learn new tasks. Each synapse is a rather complex structure in which numerous biochemical processes interact on all possible timescales. These structures are probably essential for learning, but we have currently only a few ideas about why they should be so complex. One possibility is that they are important for solving a fundamental problem in deep learning known as catastrophic forgetting. When artificial neural networks learn continually, as we do in the real world, the final performance can be surprisingly poor due to this problem. It is certainly much worse than in the case in which all the samples of the training set all available at the same time. We currently do not have a scalable solution and we cannot exclude that the solution resides in the complexity of the biological synapses. In any case, implementing a large number of synapses in a neuromorphic device is a great challenge, even when the simplest artificial synapses with only one variable are considered. This is a major limitation of the current neuromorphic systems, which either do not have the capability of learning or have a very limited ability to learn online in a complex real-world scenario with all sorts of temporal correlations.

6. What is your vision to tackle these major hurdles? Any suggestions?

**DS**: Given the high risk associated with neuromorphic computing and great device and circuit challenges, to me the key approach would be to making gradual progress starting with the simplest, yet practically useful neuromorphic systems. A good example is *ex situ* trained neuromorphic inference accelerators for deep perceptron-like and recurrent neural networks that can be used for image classification, speech recognition and related tasks, where the desired hardware impose some of the simplest requirements on the memory devices including that the device-to-device variance can be relaxed using external feedback algorithms. It is my hope that through the development of neuromorphic interference accelerators with the aim of making them competitive with their digital counterparts we will become more confident in building more complex neuromorphic computing hardware as the next steps and thus help the progression of the field. The complex systems could include but not limit to those relying on the efficient implementation of more advanced functionalities, such as synaptic plasticity in spiking neural networks or stochastic switching in Boltzmann machines, and those requiring small device-to-device variations for spiking neural networks or high switching endurance for *in situ* training.

**GI**: From the education and training point of view, one strategy is to create research programs dedicated to this emerging field particularly. The idea has started out at Caltech in the mid 90s with the Computation and Neural Systems degree program and is now also being pursued at the Institute of Neuroinformatics in the University of Zurich and ETH Zurich. While some universities have similar initiatives, most of them are spread out across multiple departments or institutions. In order to properly train a ‘neuromorphic computing scholar’ that can easily discuss, for example, about the effect of dopamine release in the frontal cortex and striatum, just as well as the effect of parasitic capacitance in circuits that comprise large transistors, I believe it is really important to have all students study and work under the same roof in one research program.

From the perspective of building efficient neuromorphic computing systems for practical or even commercial applications, my vision is to clearly identify the target application by focusing on building very specific and highly optimized computing systems (e.g., those processing sensory data in edge-computing applications), and giving up the goal to develop general purpose computing platforms.

**JG**: I think there are two possible ways. The first one is to closely mimic the brain and develop dense functionalized self-assembled computing systems. Self-assembly is a great method to build dense arrays or scaffoldings of nanodevices in two or three dimensions. It is efficient, fast and cheap. The difficulty is to power all the nanodevices and control them well enough to make them compute together. It is a promising approach but it is a long-term goal because we are far from knowing how to achieve this. The second path is to use other tricks to interconnect artificial nanoneurons and nanosynapses. Why don’t we use the communication strategies that we have developed recently to interconnect electronic devices, like wireless communication through optics or radiofrequency waves? I believe this approach is the most likely to give midterm results.

**SF**: The problem of catastrophic forgetting is one of the problems that we might be able to solve by taking inspiration from the biological brains. This is why government funding agencies like DARPA and IARPA are funding projects that contain neuroscience experiments (see e.g. the DARPA Lifelong Learning Machine (L2M) program and the IARPA Machine Intelligence from Cortical Networks (MICrONS) program). These experiments are particularly designed to focus entirely on computational principles (e.g. what is the computational advantage of complex synapses?), rather than simply describe a complex biological system. The results of these experiments might not only teach us how to design more efficient learning devices, but also reveal important principles that govern biological brains.

7. What could be the measure of when the neuromorphic computing is ready to replace the current digital computing?

**DS**: Ultimately, this will happen when, for instance, the projected performance obtained from the prototypes at small scales show compelling evidence that neuromorphic systems can outperform digital ones and/or be competitive in other metrics at the application level. Judging by the rapidly growing interest from entrepreneurs and many start-ups founded in the recent years, I would say that this might soon happen for neuromorphic inference accelerators. I also heard the possibility that dynamic vision sensor technology, which takes inspiration from biological spiking neural networks, is being close to its commercialization. This, if true, could be another milestone in neuromorphic computing. I would also like to use this opportunity to emphasize the importance of benchmarking when evaluating the potential of analog neuromorphic hardware for deep learning applications. To reach any sensible conclusions, in my opinion, rigorous comparisons of functional and physical performances across different implementations can only be conducted for well-defined applications with clear performance metrics and common benchmarks (such as classification fidelity on MNIST—a handwritten digit image set).

**GI**: This might happen much sooner than we think, and probably we will not even notice it. In the past whenever a neuromorphic circuit was deployed in a commercial product, it was never advertised or recognized as such (e.g. the Logitech Marble trackball, which used a neuromorphic motion sensor chip to measure the rotation of a ball painted with a random dot pattern, to control the cursor on a screen, see Arreguit, X. et al. *IEEE* *J. Solid State Circ*. 31, 1916−1921, 1996). I envisage that soon we will see the first instances of neuromorphic products in low-power intelligent sensors that extract information from the analog signals they measure and make simple (e.g., 1- or 2-bit) decisions without connecting to a cloud. Our cell-phones are already covered with an abundance of sensors (including pressure sensors, accelerometers, etc.), and the need to have ultra-low power always on sensory processing will eventually drive the industry to explore more exotic designs, such as those comprising analog and/or asynchronous digital neuromorphic circuits, and include them in their systems.

**JG**: I don’t think neuromorphic computing will replace digital computing. Digital computers are great at high precision computing, whilst neuromorphic computing is great for cognitive tasks such as pattern recognition, classification and prediction. Digital and neuromorphic computing will complement each other and future processors will integrate both.

8. Any suggestion on how researchers, including but not limited to material scientists, device physicists, circuits engineers, computer scientists, neuroscientists or even policy makers, can better work together in this very multidisciplinary field?

**DS**: One obvious solution to me is to organizing interdisciplinary conferences and crossing-team projects in this field. For such meetings or collaborations, it is very important that researchers are open about the problems and weaknesses of proposed approaches—not just focusing on their strengths, especially when it comes to new concepts. In my experience, however, this is often not the case and was actually why I started to conduct experiments in my research group in order to figure out many crucial yet missing details of emerging devices and circuits. This includes those ‘unwanted and raising questions’ that unfortunately could have not been disclosed in the community. For the same reason, I would like to see more critical analyses in the published papers, for instance, by providing technical arguments on why one approach would not work or one approach is better than the other. Such impartial or even negative results are not common yet, especially in the high-profile journals. I thus strongly advocate, by upfronting about the challenges and problems that we have, all the stakeholders in this field work together to build a more open, transparent and constructive environment to discuss different ideas in achieving neuromorphic engineering and computing.

**GI**: Up to now, the originally small community of neuromorphic engineers has grown and expanded thanks to the enthusiasm and dedication of its members. At the early stages (from the mid 90s to the early 2000s) when there were only a handful of groups worldwide, the Telluride Neuromorphic Engineering Workshop and the CapoCaccia Cognitive Neuromorphic Engineering Workshop were instrumental in promoting the growth of this community. Now the term “neuromorphic” has been adopted also by other communities, such as those of material scientists and device physics researchers working on memristive devices, and also started to draw lots of attentions from large industries and small startups. My hope, if not a suggestion, is that the community keeps on being open and inclusive and that its members keep on sharing tools and results with the same enthusiasm that has characterized the early years. Given the complexity of the problems tackled (e.g., to reverse-engineer the brain, to understand its computing principles by building sub-modules, or to build radically different computing platforms) there is a strong need for open collaboration and result sharing, while still leaving ample opportunities for academic and/or commercial success to all.

**JG**: It’s an important question. When I started working in the field, I didn’t want to be just developing the devices. I wanted to learn more about neuroscience, computer science and microelectronics. It is too difficult to enter a field by just reading papers. You need to talk to people. So, I have initiated a research network called GDR BioComp (http://gdr-biocomp.fr/en/description/). We organize workshops and schools about neuromorphic computing, with all our events being completely interdisciplinary. There are talks about AI, neuroscience, electronics and physics. It’s a great way to learn and the atmosphere is also great. Everybody is excited about the final common goal: making totally new brain-inspired systems. The discussions are really open and engaging because we don’t necessarily compete with each other since we are all from different fields.

**SF**: DARPA and IARPA are pioneering a new way of funding highly interdisciplinary projects that involve material scientists, physicists, the machine learning community and neuroscientists from both the industry and the academy. Other government agencies should provide more opportunities for these types of large interdisciplinary projects, possibly involving teams from multiple countries. These teams should have much more significant interaction. One of the major limitations of neuromorphic hardware is that there is a huge community of investigators who work on the algorithms for training deep networks and a separate community that works on the hardware implementations. These communities should work together. For example, those who design the electronic circuits often assume that there is a standard algorithm they need to implement and they make all possible efforts to do it efficiently. This is actually not the case. Very often, it is possible to achieve the same performance using a different algorithm that is much easier to implement and can even take advantage of the peculiarities of the materials chosen. This approach would be very much in the spirit of what Carver Mead proposed more than 30 years ago. It is important to take advantage of the physics of the electronic devices that we’re going to use and it is often possible to do it more efficiently by modifying the algorithms we implement.

*The interview was done by Nature Communications editors Congcong Huang, Selina La Barbera and Cephas Small*.

